# Role of airway epithelial cell miRNAs in asthma

**DOI:** 10.3389/falgy.2022.962693

**Published:** 2022-09-20

**Authors:** Eistine Boateng, Draginja Kovacevic, Vladimira Oldenburg, Madeleine Rådinger, Susanne Krauss-Etschmann

**Affiliations:** ^1^ Early Life Origins of Chronic Lung Disease, Research Center Borstel, Leibniz Lung Center, Airway Research Center North (ARCN), German Center for Lung Research (DZL), Borstel, Germany; ^2^ DZL Laboratory for Experimental Microbiome Research, Research Center Borstel, Leibniz Lung Center, Airway Research Center North (ARCN), German Center for Lung Research (DZL), Borstel, Germany; ^3^ Krefting Research Centre, Department of Internal Medicine and Clinical Nutrition, Institute of Medicine, University of Gothenburg, Gothenburg, Sweden; ^4^ Institute for Experimental Medicine, Christian-Albrechts-Universität zu Kiel, Kiel, Germany

**Keywords:** asthma, miRNA, signaling, epithelial cells, lung

## Abstract

The airway epithelial cells and overlying layer of mucus are the first point of contact for particles entering the lung. The severity of environmental contributions to pulmonary disease initiation, progression, and exacerbation is largely determined by engagement with the airway epithelium. Despite the cellular cross-talk and cargo exchange in the microenvironment, epithelial cells produce miRNAs associated with the regulation of airway features in asthma. In line with this, there is evidence indicating miRNA alterations related to their multifunctional regulation of asthma features in the conducting airways. In this review, we discuss the cellular components and functions of the airway epithelium in asthma, miRNAs derived from epithelial cells in disease pathogenesis, and the cellular exchange of miRNA-bearing cargo in the airways.

## Introduction

The lung is constantly exposed to particles with diverse aerodynamic properties that determine where they are deposited in the airways. The airway epithelial cells exhibit a barrier function to protect the submucosal layer from pathogens, allergens, and pollutants in inhaled air. Furthermore, the airway epithelium coordinates with cells of the innate and adaptive immune systems to maintain host defense. In allergic disease, airway epithelial cells produce cytokines e.g. TSLP, IL-25, and IL-33 which drives dendritic cells toward Th2 response, activates eosinophils, and stimulates the production of type 2 cytokines in mast cells, respectively (1–4). Thus, epithelial cells support the initiation of airway inflammation and the pathogenesis of allergy related diseases e.g. allergic asthma.

Asthma is a complex, heterogenous disease characterized by symptoms such as recurrent episodes of wheezing, breathlessness, chest tightness, cough, and expiratory airflow fluctuation. Asthma is often dichotomized into endotypes distinguished by pathophysiological mechanisms. The most studied endotypes are type 2 and non-type 2, which are defined by various phenotypes (5). Emerging studies are still unraveling the pathophysiological mechanisms of asthma (5–8) though it is known that the disease is marked by chronic airway inflammation (9) which subsequently leads to hypersecretion of mucus, bronchial hyperresponsiveness, and airway edema and remodeling (7, 10, 11). Airway epithelial remodeling in asthma is demonstrated by subepithelial fibrosis, thickening of the sub-basement membrane, increase in smooth muscle mass, mucus gland hypertrophy, and vascular congestion, culminating in airway congestion and bronchoconstriction (6, 12). These prominent pathological features highlight the involvement of the airway epithelium in asthma [reviewed in (13, 14)]. Therefore, the epithelial biology of the conducting airways fundamentally provides insight to the understanding of the mechanistic development of asthma.

Goblet cell hyperplasia and mucus hypersecretion are striking pathophysiological features reported during asthma exacerbation (15). To define the molecular mechanisms leading to these manifestations, some studies have explained the role of EGFR in mucus cell metaplasia and mucus production in bronchial epithelial cells (16–18). Furthermore, a study done in patients with asthma demonstrated that epithelial cells increase the levels of GAD65/67 and GABA_A_R subunits in response to allergen challenge (19). The authors showed that the inhibition of GABA signaling decreased ovalbumin or IL-13-induced goblet cell hyperplasia and mucin production in mice.

It is reported that epithelial cells express several asthma susceptibility genes e.g. *IL-1RL1, IL-33, TSLP, CDHR3, PCDH1, MUC5AC, KIF3A, EFHC1* and *GSDMB* (11, 20–22). Moreover, epithelial gene expression can be modulated by miRNAs, small noncoding RNAs that bind to target mRNAs leading to degradation or even translational repression (23–25). miRNAs are ∼22 nucleotides in length (23), and processed by two RNase III proteins, Drosha and Dicer. After transcription, the miRNA, known as primary or pri-miRNA, is processed into a precursor miRNA (pre-miRNA) and further into mature miRNA. It is widely known that miRNAs bind to sequence-specific recognition sites at the 3′ UTR of their target to repress gene expression. Nonetheless, miRNAs also bind to regions such as the 5′ UTR, coding sequence, and promoters (26). The molecules not only suppress genes, but also stimulate gene expression (27). miRNAs are involved in numerous biological processes, including the pathogenesis of many diseases. Therefore, miRNAs could serve as valuable biomolecules for disease diagnosis, progression, exacerbation and treatment response. For instance, as miRNAs are dysregulation during the pathogenesis of allergy and asthma, their levels can be quantitatively explored in patient samples from the skin, lung, and nose, and in sputum, exhaled breath condensates, and specific cell types (28).

miRNAs have been shown to be differentially expressed in bronchial epithelial cells following diesel exhaust particle, tobacco smoke, and virus exposures (28). Indeed, several miRNAs modulate genes involved in epithelial barrier function, proliferation, response to viral infection and mucus production (29–33). It is however suggest that the impact of miRNAs on the airway epithelium might differ with disease severity (31).

The thickening of the epithelium (34) and induction of goblet cell hyperplasia in asthma suggest a role for a dysregulated network of intracellular molecules in response to alterations in the milieu of the lung. We have earlier discussed the engagement of miRNAs in multiple gene regulation during the pathogenesis of asthma (35). miRNAs are altered in bronchial epithelial cells in patients with asthma (32), and some evidence suggests their contribution to the modulation of genes controlling cellular phenotypes. For example, a study showed that in humans, the expression of miR-4423 was restricted in the airway epithelium, while the overexpression of this miRNA in normal human bronchial epithelial cells (HBECs) contributed to an increase in cells expressing ciliated cell markers (36). Additionally, there was defective multiciliogenesis in the trachea, bronchus, and bronchiole of miR-34/449 “triple knockout” embryos (37), suggesting that the miR-34/449 family is essential for normal ciliation in the respiratory epithelium. Another study also used normal HBECs to demonstrate that miRNAs are differentially expressed in differentiated and undifferentiated cells (38). Other biological processes have been shown to be regulated by miRNAs in the lung epithelium, including the disruption of intercellular adhesion proteins and release of cytokines (39).

The protective function of epithelial cells in the airway is periodically challenged, as they are the first point of contact from the environment. The epithelia of the proximal and distal air spaces are variously vulnerable depending on the velocity of air entering the lungs. Hence, the airway epithelium is a primary site for the commencement and aggravation of respiratory diseases. In this review, we summarize the cellular components of the airway epithelium and their roles in the pathogenesis of asthma. Importantly, we focus on airway epithelial cell function in asthma, the connection between deregulated miRNAs and epithelial cell function, and finally, cross-talk in the microenvironment of the airway epithelium.

### The airway epithelium

The microscopic aspects of lung development and signaling mechanisms have been exquisitely explained (40). As this review is about asthma, our focus is particularly on the lower respiratory system, which consists of the conducting and respiratory zones. The trachea, bronchi, and terminal bronchioles make up the conducting zone. The respiratory bronchioles, alveolar duct and alveoli form the respiratory zone, consisting of different epithelial cell types. The conducting zone transports respiratory gases and the respiratory zone functions in gas exchange. The respiratory epithelium comprises pseudostratified ciliated columnar epithelium with other functionally heterogeneous cells. The diverse cellular components include goblet, ciliated columnar, basal, brush, neuroendocrine cells, and pulmonary ionocytes. The airway epithelium merges with the pulmonary alveolar epithelium which consists of type I and type II cells. The type I cells make up the majority of the alveolar surface area and are essential for the air-blood barrier in the lungs (41, 42). The type II cells differentiate into type I cells in response to injury repair (43, 44).

In response to injury and stress, the lung is capable of regenerating the airway epithelium. The multipotent capacity of a specific cell type in the neighborhood of the basal lamina defines the cellular lineage hierarchy in the bronchial airway. These cells, known as basal cells, act as progenitor cells that maintain airway homeostasis through self-renewal and differentiation into specialized cell types such as ciliated and goblet cells (45, 46). Though it is unclear which signaling pathways primarily orchestrate the process, basal cells are capable of differentiating into tuft cells, pulmonary ionocytes, and neuroendocrine cells (47). In contrast, it is well documented that the fate of basal cell differentiation into other cell types is mediated by transcriptional networks of the Notch signaling pathway (48). Despite the multipotent progenitor characteristics of basal cells, their abundance is diverse in the conducting airways. In the human lung, basal cells are higher in the large airways as compared to the smaller airways (49). In addition to basal cells, maintenance of the airway epithelium is also supported by some subsets of club cells that terminally differentiate into ciliated cells in response to epithelial damage (50, 51). Additionally, pulmonary neuroendocrine cells in the bronchial epithelium proliferate and repair the neighboring epithelium (52), and rare clusters are shown to function as stem cells (53).

The ciliated cells of the epithelium have numerous motile cilia protruding from basal bodies on the apical part of the cells. These cilia are made of structural and motor proteins that orchestrate coordinated ciliary movement in the airway. Thus, the protective layer of mucus lining the epithelium is biomechanically beaten by the cilia in the direction leading outside the lung. This function supports the removal of inhaled particles and pathogens from the airways. It is suggested that motile cilia in the respiratory epithelium possess various forms of sensory functions such as mechanoreceptive and chemoreceptive properties [reviewed in (54)]. In contrast to their biomechanical role, ciliated cells of the airway are also targets for invading bacteria and viruses (55). Additionally, it has been demonstrated that ciliated cells transdifferentiate into specialized epithelial cells in response to injury (56).

Club cells are cuboidal non-ciliated, non-mucus secreting, and microvilli studded, with dense cytoplasmic granules (57). These cells are dome-shaped, facing the bronchiolar lumen. In humans, club cells are heterogeneous, and one subtype expresses high levels of the basal cell marker KRT5 (58). Like basal cells, this population of club cells is multipotent (58). There are variants of functional club cells located in the airways. For example, at the bronchioalveolar-duct junction and the microenvironment of neuroendocrine cells, club cells can assume progenitor properties to support epithelial regeneration in response to injury (59–61). Other functional features of club cells include their critical role in inflammation, biotransformation of environmental toxicants, mitigation of oxidative stress, and maintenance of the barrier function of the airway epithelium (62–64). Participation in these mechanisms involve the secretion of club cell protein 10 (CC10), KL-6 protein, lipids, glycoproteins, and surfactants (surfactant proteins A, B, and D).

Goblet cells are located throughout the conducting airway of the respiratory epithelium. Ultrastructural analysis of these cells shows that they are polarized, with nuclei located on the basal side and secretary granules located on the apical side. Along with club cells, goblet cells are also formed from epithelial cells under the triggering influence of IL-13 (65). In addition to IL-13, some inflammatory mediators, irritant gases, proteinases, bacterial products, and cigarette smoke exposures increase the number of goblet cells in the airways (66). Goblet cells contribute mucins to the mucus component in the airways, supporting the viscoelastic property of the mucus in the airway.

Since their discovery, the physiological and mechanical roles of pulmonary ionocytes were associated with mucus viscosity and clearance in cystic fibrosis (47, 67). Pulmonary ionocytes are differentiated from basal cells (47, 67) and transcriptionally distinguished by the expression of *FOXI1* and the highest level of *CFTR* among the cells in the airway epithelium (68). Apart from basal cell differentiation into ionocytes, a possible cell lineage from tuft-like cells has been shown (69). In humans, *FOXI1*^+^ ionocytes are found in reduced amounts in the small, compared to large airway epithelium (68). Also, antibody inhibition of the Notch receptors in HBECs has been shown to decrease the number of pulmonary ionocytes (67), suggesting the influence of the signaling pathway in their production.

### Epithelial cells in asthma

As a first line of defense, the mucus lining of the epithelium traps foreign particles for further clearance. Usually after an invasion by particles from the environment, epithelial cells react by increasing mucus secretion and ciliary beating in an attempt to cleanse the airway of harmful and insoluble particles. Disruptions in the mucus barrier expose the epithelial cells to allergens and noxious agents which could lead to severe damages. However, epithelial cells are strongly linked together by tight and adherens junctions to form a protective physical barrier over the underlying stromal region of the airway. Thus, the integrity of the airway epithelial landscape and the junctional complexes is essential to cushion against excessive injury from particles and invading pathogens.

Epithelial cells are often frail in asthmatic airways. This is marked by apoptosis and shedding of epithelial cells in the bronchial mucosa of asthma patients compared to healthy controls (70). In an *in vitro* experiment, allergen exposed airway epithelial cells released IL-25 (71). In another study on patients with mild atopic asthma, the epithelium and submucosa of bronchial biopsies showed increased immunoreactivity to IL-25, IL-33, and TSLP after allergen challenge (72). These findings highlight the role of airway epithelial cells in supporting inflammation by synthesizing lipid mediators and cytokines, which contribute to some of the pathological features of asthma (1, 73). As part of the initial steps in allergic response to inhaled particles and pathogens, the airway epithelial cells release chemokines and cytokines to attract and activate dendritic cells. These cytokines also activate the group 2 innate lymphoid cells (ILC2s) known to support type 2 inflammation in allergic diseases (74). Altogether, the interplay between airway epithelial cells and antigen-presenting cells in the milieu of inflammatory mediators stimulate key features of asthma.

Airway epithelial cells express various proportions of molecules that support the pathogenesis of asthma and indicate susceptibility to the disease. In an experimental mouse model, the Toll-like receptor (TLR) 4 was found to be highly expressed in the conducting airway epithelial cells (75). In this study, inhibition of TLR4 reduced airway lymphocytosis and eosinophilia, IL-5 and IL-13 in bronchoalveolar lavage fluid (BALF), peri-bronchovascular infiltrates, goblet cell hyperplasia, and airway hyperresponsiveness in HDM treated mice. There is also evidence that the cadherin-related family member 3 *(CDHR3)* expressed in airway epithelial cells is associated with exacerbation susceptibility in children with severe asthma (76). Another molecule, protocadherin 1 *(PCDH1)*, expressed in bronchial epithelial cells, has emerged as a susceptibility gene for bronchial hyperresponsiveness and asthma in children (77, 78). In addition, these genes which are expressed in epithelial cells also indicate asthma susceptibility: *ADAM33*, *GPRA*, *SPINK5*, *IRAKM*, *DPP10*, and *HLA-G* (79).

The specialized functions of the cells in the airway epithelium suggest their independent response in allergy and asthma conditions. These cells encounter various levels of molecular-specific alterations detrimental to their functional performance. In a recent study, the level of TRPV4 was demonstrated to be increased in the apical membrane of bronchiolar cells in biopsies from asthma patients (80). Elevated levels of TRPV4 in club cells were moreover shown to act as signals for allergic inflammation. Previous studies have also demonstrated significantly reduced CC10 in the epithelial cells of small airways and in the plasma of asthma patients compared to controls (81, 82). A decrease in serum levels of CC10 has also been reported in patients with overlapping features of asthma and COPD (83). In mouse model of OVA-induced asthma, the mRNA and protein levels of CC10 were reduced in lung tissues and the airway, respectively (84), and CC10 was further associated with exacerbation of asthmatic phenotypes. These studies show that the immunomodulatory, anti-inflammatory, and anti-phospholipase A2 properties of CC10 are threatened in asthma conditions, suggesting a functional role of club cells in the disease.

A characteristic feature of airway epithelial remodeling is goblet cell metaplasia. In a mouse model with conditionally expressed *spdef* in the non-ciliated epithelial cells, increases were found in goblet cell metaplasia, eosinophil infiltration, and airway hyperresponsiveness (85). Again, there were marked increase in the mRNA levels of Th2 cytokines (*Il-13* and *Ccl17)*, eosinophilic chemoattractants (*Ccl24* and *Ccl20)* and targets that signify dendritic cell activation (*Il-33*, *Il-25*, and *Csf2)* (85). Noteworthy, goblet cell metaplasia is coordinated by a wide range of molecules and signaling pathways e.g. Notch2, IL-4, IL-13, TNF-α, and EGF (86–89). In both mice and cultured epithelial cells, inhibition of IL-13 and the STAT6 signaling attenuated goblet cell metaplasia (90). In the mice, there was also evidence of decreased goblet cell metaplasia and prevention of HDM-induced airway hyperreactivity and inflammation. In another mouse study, inhibition of the CREB binding protein (CBP)/*β*-catenin pathway mitigated goblet cell metaplasia and mucus production in an HDM-induced model of allergic airway disease (91). Elevated FOXM1 has been shown in goblet and club cells of an HDM asthma mouse model and in patients with severe asthma (92). Deletion of FOXM1 in club cells decreased inflammation and airway resistance in an HDM-induced mouse model, and also reduced goblet cell metaplasia in the airway epithelium (92). Hyperplasia of goblet cells is also associated with chronic hypersecretion of mucus in asthma. The mechanisms of goblet cell hyperplasia and contribution to the apical mucus layer of the epithelium in humans and asthma mouse models have been described elsewhere (15, 93–96).

Ciliary function remains an important component of the respiratory airway epithelium. Despite the chemical barrier functions elicited by the airway epithelial cells the biomechanical action of ciliated cells is relevant for the clearance of foreign particles. Airway epithelial cell cilia respond to chemical stimuli and the physical properties of liquid components overlying them (97–99). Epithelial cell loss is usually marked in asthmatic airways – instigated by disruption and decreased formation of cellular tight junctions (100). These epithelial changes increase permeability to allergens (100, 101), mucus hypersecretion, and depletion of ciliated cells in the airway (102). Progenitor cells expressing *FOXJ1*, a marker for ciliated cells, are capable of differentiating into goblet cells under the influence of IL-13 (103). This underscores the role of ciliated cells in epithelial regeneration in response to injury. In bronchial biopsies from children with chronic onset of asthma, some cells co-expressed mucus (*MUC5AC* and *CEACAM5*) and ciliated cell genes (*FOXJ1* and *PIFO*), and were named as mucus ciliated cells (104). It was further demonstrated that the cells exhibited elevated levels of IL-4/IL-13-induced genes, as well as asthma genes such as *CSTI* and *POSTN*, suggesting the involvement of these types of cells in mucus cell metaplasia (104).

### Epithelial cell miRNAs in asthma

The role of airway epithelial cells in the pathogenesis of asthma merit the discussion of intracellular molecules that are functionally active in disease progression or symptom alleviation. miRNAs are dysregulated in respiratory diseases, and capable of modulating the expression of various genes implicated in airway biology and diseases (35). Murine models support investigations into miRNAs and their role in asthma and allergic airway disease. Using HDM treatment as a model for allergic airway disease in mice, data from one study showed greater than 5-fold increase in miR-145, miR-21 and let-7b in dissected airways compared to samples from saline-treated mice (105). Notably, inhibition of miR-145 reduced HDM-induced mucus hypersecretion in the airway epithelial cells and recruitment of eosinophils to the airway (105). Eosinophil recruitment was further demonstrated by a reduced number of eosinophils in BALF. However, the authors neither identified specific targets nor described the molecular mechanisms leading to reduced mucus hypersecretion after miR-145 antagonism.

In response to HDM-induced allergic asthma, one study found elevated levels of miR-16, miR-21, and miR-126 in the airway walls of mice (106). On the contrary, HDM-induced TLR-4 and MyD88 deficient mice demonstrated decreased levels of one of the miRNAs i.e. miR-126. Inhibition of miR-126 in the airway wall of WT mice suppressed HDM-induced mucus hypersecretion and other allergy-related features (106). Moreover, the MyD88 signaling pathway was found to regulate the expression of miR-126 and HDM-induced allergic inflammation. Altogether, transcriptomes of the airway walls of HDM challenged mice treated with ant-miR-126 showed differential regulation of genes that encode for proteins making up certain regions of the Ig kappa, Ig lambda and heavy chains. OBF.1 [Oct binding factor 1 a.k.a. BOB.1 (B-cell Oct binding protein 1)] and PU.1 were significantly upregulated, whereas GATA3 was downregulated. OBF.1 regulated the production of antibodies and PU.1 negatively regulated TLR4 and Th2 response.

Anti-mmu-mir-106a attenuated goblet cell metaplasia and mucin content in the airways of mice with OVA-induced allergic asthma (107). However, the molecular events leading to reduced goblet cell metaplasia require further investigation, as this was not demonstrated in the study. In contrast, some studies have demonstrated the involvement of miR-125b and miR-330 in the inhibition of goblet cell differentiation in allergic airway inflammation and in IL-13-induced MUC5AC secretion in bronchial epithelial cells, respectively (108, 109). Intranasal administration of miR-145–5p antagomir to mice reversed HDM-induced decrease in KIF3A (expressed in airway epithelial cells), reduced inflammatory response in the lung, and ultimately, modulated asthma phenotypes (39).

Analyses of samples from human subjects have been instrumental in studying airway biology and miRNAs in asthma. Dysregulated levels of miRNAs are reported in diverse human samples. For instance, decreased levels of miR-34b-5p, miR-34c-5p, miR-449a and miR-449b-5p were demonstrated in bronchial epithelial brushings from both steroid-naive and steroid-using asthma cohorts (110). As already described, miR-449 is important for the differentiation of ciliated cells (111). This was demonstrated by overexpressing miR-449 in proliferating human airway epithelial cells (HAECs), which eventually decreased the levels of NOTCH1 and DLL1. Additionally, inhibition of miR-449 in differentiating HAECs increased the protein levels of NOTCH1 and DLL1. Altogether, lower protein levels of NOTCH1 and DLL1 were found in multiciliated cells compared to undifferentiated and non-ciliated differentiated cells (111). Since the pathogenesis of asthma is impacted by multiple genetic factors, the reduction of miR-34b-5p and miR-34c-5p in asthma should be further investigated to determine their molecular influence in the disease.

Microarray analyses of bronchial epithelial cells from asthmatic patients have also shown decreased levels of miR-18a, miR-27a, miR-128, and miR-155 (32). Inhibition of all of these miRNAs together increased the release of IL-6 from bronchial epithelial cells compared to inhibition of each miRNA individually. Importantly, these findings potentially caution the selection of specific dysregulated miRNAs to provide mechanistic explanations of phenotypes and pathological observations in experimental disease conditions.

Bronchial epithelial cells from mild asthmatic patients indicated strong downregulation of miR-203 and significant increases in miR-487b, miR-181c, and miR-let-7f levels (112). Higher expression of the aquaporin gene *AQP4*, a predicted target for miR-203, was found in bronchial epithelial cells from mild asthma patients. *AQP4* controls water transport across the cell membrane and we suggest that the level is possibly upregulated in the bronchial epithelium to support the clearance of obstructive fluids in asthmatic airways. Hence, miR-203 is decreased to support this process in asthma pathogenesis.

Among the numerous miRNAs differentially expressed in the bronchial epithelium of healthy and severe asthma patients, the levels of miR-19b-3p, miR-20a-5p, and miR-135b-5p were shown to be decreased while those of miR-574-3p and miR-625-3p were increased in asthma patients after validation in an expanded cohort (113). Using the Ingenuity Pathway Analysis, the authors ascertained that the numerous miRNAs dysregulated in severe asthma condition relate to several biological processes including epithelial repair and inflammation (113).

In a recent study, miR-141-3p was increased in primary HBECs and as well induced following allergic stimulation in patients with asthma (33). Knockdown of miR-141-3p in IL-13-treated HBECs led to decreased goblet cell genes and mucus-producing goblet cells (33). Also, endobronchial biopsy samples from patients with severe asthma displayed increased epithelial thickness, and bronchial epithelial cells showed an elevated rate of cell proliferation compared to samples from patients with mild asthma (30). Corresponding with the observation, the level of miR-19a was increased in bronchial epithelial cells in the severe asthma condition. Further evidence revealed that the transfection of miR-19a into bronchial epithelial cells promoted cell proliferation (30). Airway biopsies of patients with asthma demonstrated a decrease in miR-146a compared to the level in non-asthmatic controls (114). Additionally, stimulation of HBECs with cytokines associated with asthma (TNF-α, IL-17A, and IL-4) resulted in increased miR-146a. Thus, miR-146a response to pro-inflammatory cytokines implicates its relevance in neutrophilic asthma (114). Moreover, in this study, patients with neutrophilic asthma showed significant increase in the mRNA levels of IL-8 and CXCL1 in airway epithelial cells. Importantly, a negative association between miR-146a level (in bronchial brushing) and neutrophil counts (in BALF) was reported in asthmatic patients (114). The authors showed that overexpression of miR-146a in HBECs controlled inflammatory response by reducing the mRNA level and release of IL-8 and CXCL1, respectively. Additionally, the mRNA levels of the interferon response genes, *IFITM1* and *IRF1*, were moderately increased in miR-146a transfected HBECs. Nevertheless, the regulation of these proteins and genes in this study only indicates a causal effect from miR-146a transfection into HBECs and not necessarily the regulation of putative targets.

**Figure 1:**
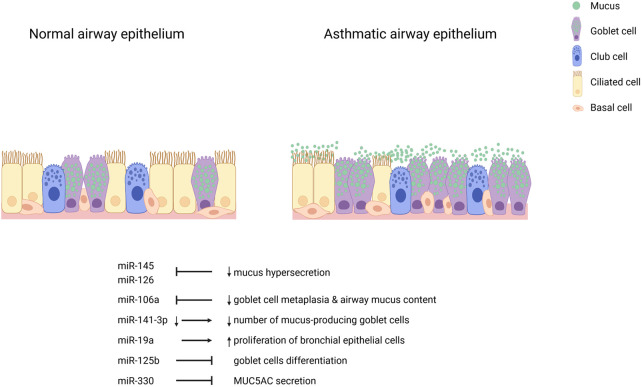
Airway epithelial cell miRNAs in asthma. Inhibition of miR-145 and miR-126 decrease mucus hypersecretion. Inhibition of miR-106a reduces goblet cell metaplasia and airway mucus content. Decreased miR-141-3p leads to fewer mucus-producing goblet cells. miR-19a induces the proliferation of bronchial epithelial cells. miR-125b and miR-330 inhibit goblet cell differentiation and IL-13-induced MUC5AC secretion, respectively.

While miRNAs regulate various genes to modulate phenotypes in the airway epithelium of asthma patients and experimental conditions, a single miRNA can also regulate multiple genes and several miRNAs can regulate the same gene. The epithelium of patients with asthma display several structural and functional anomalies linked to dysregulated miRNAs. miRNA candidates profiled in the airway epithelium may work synergistically or elicit additive effects to other subsets in the microenvironment of the epithelium to modulate these phenotypes. Thus, we posit that the study of specific miRNAs among a dysregulated pool may not adequately disentangle the mechanistic involvement of epithelial miRNAs in the pathogenesis of asthma. Because miRNA dysregulation in the asthmatic airway epithelium may be molecularly programmed in response to triggers, miRNAs should be collectively studied in relation to phenotypes in asthmatic airways and the pathogenesis of the disease in general. Figure 1 shows airway epithelial cell miRNAs and the features of asthma they are mechanistically involved with.

### Cellular cross-talk in the vicinity of the airway epithelium

Cells release extracellular vesicles (EVs) containing bioactive cargoes that influence cellular functions in asthma (115, 116). Thus, the exchange of biomolecules in the vicinity of the airway epithelium may be supported by EVs. In a recent publication, it was demonstrated that epithelial cells exchange cargoes to alter mucin hypersecretion and miRNA content of exosomes in target cells (117).

EV miRNAs are believed to support the infiltration of inflammatory cells to the lungs, regulate Th2 inflammation, modulate mast cell degranulation, regulate cytokine production, and function in airway remodeling and hyperresponsiveness (118). Under physiological and pathological conditions, the number and content of EVs is altered. For example, treatment of HBECs with T2 (IL-4 + IL-13) and T17 (IL-17A + TNFα) cytokines induced the release of EVs (119). In an in-vitro model of experimental asthma, dysregulated miRNAs were found in the EV cargoes of normal HBECs challenged with IL-13 (120). These findings suggest a link between miR-34a, miR-92b and miR-210 to early stage Th2 response in the airway and asthma. In another study, HDM-exposed mice showed increased BALF EVs bearing miRNAs suggested to be involved in allergic airway inflammation (121).

Dysregulated intracellular miRNAs in airway epithelial cells determine cellular response and phenotypes. The cross-talk orchestrated through cargo exchange between the cells is therefore a key component in asthma pathogenesis. Moreover, cell signaling mechanisms and pathways crucial for the progression and mitigation of asthma require the unraveling of miRNAs generated by specific cells in the airways. This could support the understanding of the pathomechanisms of goblet cell hyperplasia and metaplasia, and mucus hypersecretion in asthma.

## Conclusion

Epithelial cells are the first cellular contact of environmental materials entering the lung. miRNA regulation of targets in the airway support the maintenance of epithelial barrier integrity or dysfunction in asthma. Communications established between epithelial cells in the airways depict the relevance of miRNAs in response to alterations in the cellular milieu in asthmatic conditions. A gap of knowledge remains when it comes to understanding the role of epithelial cell miRNAs in asthma. Further studies require the use of single cell small RNA sequencing combined with functional assays, including cell lines with genetically modified miRNAs and target genes to expand this research field. Moreover, defining the possible vesicular cargo exchanges between the epithelial cells in the airways and dysregulated miRNAs in each specific cell type is essential to broaden understanding of signaling pathways connected to the pathophysiology of asthma.

## References

[1] Ying S O'Connor B Ratoff J Meng Q Mallett K Cousins D Thymic stromal lymphopoietin expression is increased in asthmatic airways and correlates with expression of Th2-attracting chemokines and disease severity. J Immunol. (2005) 174(12):8183–90. 10.4049/jimmunol.174.12.8183 15944327

[2] Zhou B Comeau MR De Smedt T Liggitt HD Dahl ME Lewis DB Thymic stromal lymphopoietin as a key initiator of allergic airway inflammation in mice. Nat Immunol. (2005) 6(10):1047–53. 10.1038/ni1247 16142237

[3] Altman MC Lai Y Nolin JD Long S Chen CC Piliponsky AM Airway epithelium-shifted mast cell infiltration regulates asthmatic inflammation via Il-33 signaling. J Clin Invest. (2019) 129(11):4979–91. 10.1172/JCI126402 31437129 PMC6819127

[4] Suzukawa M Morita H Nambu A Arae K Shimura E Shibui A Epithelial cell-derived Il-25, but not Th17 cell-derived Il-17 or Il-17f, is crucial for murine asthma. J Immunol. (2012) 189(7):3641–52. 10.4049/jimmunol.1200461 22942422 PMC3812057

[5] Kuruvilla ME Lee FE Lee GB. Understanding asthma phenotypes, endotypes, and mechanisms of disease. Clin Rev Allergy Immunol. (2019) 56(2):219–33. 10.1007/s12016-018-8712-1 30206782 PMC6411459

[6] Gans MD Gavrilova T. Understanding the immunology of asthma: pathophysiology, biomarkers, and treatments for asthma endotypes. Paediatr Respir Rev. (2020) 36:118–27. 10.1016/j.prrv.2019.08.002 31678040

[7] Saetta M Turato G. Airway pathology in asthma. Eur Respir J Suppl. (2001) 34:18s–23s. 10.1183/09031936.01.00229501 12392031

[8] Suraya R Nagano T Katsurada M Sekiya R Kobayashi K Nishimura Y. Molecular mechanism of asthma and its novel molecular target therapeutic agent. Respir Investig. (2021) 59(3):291–301. 10.1016/j.resinv.2020.12.007 33549541

[9] Reddel HK Bateman ED Becker A Boulet LP Cruz AA Drazen JM A summary of the new gina strategy: a roadmap to asthma control. Eur Respir J. (2015) 46(3):622–39. 10.1183/13993003.00853-2015 26206872 PMC4554554

[10] Bergeron C Tulic MK Hamid Q. Airway remodelling in asthma: from benchside to clinical practice. Can Respir J. (2010) 17(4):e85–e93. 10.1155/2010/318029 20808979 PMC2933777

[11] Heijink IH Kuchibhotla VNS Roffel MP Maes T Knight DA Sayers I Epithelial cell dysfunction, a major driver of asthma development. Allergy. (2020) 75(8):1902–17. 10.1111/all.14421 32460363 PMC7496351

[12] Mims JW. Asthma: definitions and pathophysiology. Int Forum Allergy Rhinol. (2015) 5(Suppl 1):S2–S6. 10.1002/alr.21609 26335832

[13] Calven J Ax E Radinger M. The airway epithelium-a central player in asthma pathogenesis. Int J Mol Sci. (2020) 21(23):8907. 10.3390/ijms21238907 PMC7727704 33255348

[14] Wang Y Bai C Li K Adler KB Wang X. Role of airway epithelial cells in development of asthma and allergic rhinitis. Respir Med. (2008) 102(7):949–55. 10.1016/j.rmed.2008.01.017 18339528

[15] Rogers DF. Airway goblet cell hyperplasia in asthma: hypersecretory and anti-inflammatory? Clin Exp Allergy. (2002) 32(8):1124–7. 10.1046/j.1365-2745.2002.01474.x 12190646

[16] Casalino-Matsuda SM Monzon ME Forteza RM. Epidermal growth factor receptor activation by epidermal growth factor mediates oxidant-induced goblet cell metaplasia in human airway epithelium. Am J Respir Cell Mol Biol. (2006) 34(5):581–91. 10.1165/rcmb.2005-0386OC 16424381 PMC2644222

[17] Enomoto Y Orihara K Takamasu T Matsuda A Gon Y Saito H Tissue remodeling induced by hypersecreted epidermal growth factor and amphiregulin in the airway after an acute asthma attack. J Allergy Clin Immunol. (2009) 124(5):913–20 e1-7. 10.1016/j.jaci.2009.08.044 19895983

[18] Jia Z Bao K Wei P Yu X Zhang Y Wang X Egfr activation-induced decreases in Claudin1 promote Muc5ac expression and exacerbate asthma in mice. Mucosal Immunol. (2021) 14(1):125–34. 10.1038/s41385-020-0272-z 32132671

[19] Xiang YY Wang S Liu M Hirota JA Li J Ju W A gabaergic system in airway epithelium is essential for mucus overproduction in asthma. Nat Med. (2007) 13(7):862–7. 10.1038/nm1604 17589520

[20] Bhaker S Portelli MA Rakkar K Shaw D Johnson S Brightling C Human bronchial epithelial cells from patients with asthma have an altered gene expression profile. ERJ Open Res. (2022) 8(1):00625–2021. 10.1183/23120541.00625-2021 35198626 PMC8859501

[21] Tsai YH Parker JS Yang IV Kelada SNP. Meta-analysis of airway epithelium gene expression in asthma. Eur Respir J. (2018) 51(5):1701962. 10.1183/13993003.01962-2017 29650561 PMC7395676

[22] Woodruff PG Boushey HA Dolganov GM Barker CS Yang YH Donnelly S Genome-wide profiling identifies epithelial cell genes associated with asthma and with treatment response to corticosteroids. Proc Natl Acad Sci U S A. (2007) 104(40):15858–63. 10.1073/pnas.0707413104 17898169 PMC2000427

[23] Bartel DP. Metazoan micrornas. Cell. (2018) 173(1):20–51. 10.1016/j.cell.2018.03.006 29570994 PMC6091663

[24] Johansson K Weidner J Radinger M. Micrornas in type 2 immunity. Cancer Lett. (2018) 425:116–24. 10.1016/j.canlet.2018.03.036 29604393

[25] Mehta A Baltimore D. Micrornas as regulatory elements in immune system logic. Nat Rev Immunol. (2016) 16(5):279–94. 10.1038/nri.2016.40 27121651

[26] Broughton JP Lovci MT Huang JL Yeo GW Pasquinelli AE. Pairing beyond the seed supports microrna targeting specificity. Mol Cell. (2016) 64(2):320–33. 10.1016/j.molcel.2016.09.004 27720646 PMC5074850

[27] Vasudevan S. Posttranscriptional upregulation by micrornas. Wiley Interdiscip Rev RNA. (2012) 3(3):311–30. 10.1002/wrna.121 22072587

[28] Weidner J Bartel S Kilic A Zissler UM Renz H Schwarze J Spotlight on micrornas in allergy and asthma. Allergy. (2021) 76(6):1661–78. 10.1111/all.14646 33128813 PMC8246745

[29] Bondanese VP Francisco-Garcia A Bedke N Davies DE Sanchez-Elsner T. Identification of host mirnas that may limit human rhinovirus replication. World J Biol Chem. (2014) 5(4):437–56. 10.4331/wjbc.v5.i4.437 25426267 PMC4243148

[30] Haj-Salem I Fakhfakh R Berube JC Jacques E Plante S Simard MJ Microrna-19a enhances proliferation of bronchial epithelial cells by targeting Tgfbetar2 gene in severe asthma. Allergy. (2015) 70(2):212–9. 10.1111/all.12551 25443138

[31] Huang H Lu H Liang L Zhi Y Huo B Wu L Microrna-744 inhibits proliferation of bronchial epithelial cells by regulating Smad3 pathway via targeting transforming growth factor-Beta1 (Tgf-Beta1) in severe asthma. Med Sci Monit. (2019) 25:2159–68. 10.12659/MSM.912412 30903795 PMC6441316

[32] Martinez-Nunez RT Bondanese VP Louafi F Francisco-Garcia AS Rupani H Bedke N A microrna network dysregulated in asthma controls Il-6 production in bronchial epithelial cells. PLoS One. (2014) 9(10):e111659. 10.1371/journal.pone.0111659 25360780 PMC4216117

[33] Siddiqui S Johansson K Joo A Bonser LR Koh KD Le Tonqueze O Epithelial Mir-141 regulates Il-13-induced airway mucus production. JCI Insight. (2021) 6(5):e139019. 10.1172/jci.insight.139019 PMC8021117 33682796

[34] Cohen LEX Tarsi J Ramkumar T Horiuchi TK Cochran R DeMartino S. Epithelial cell proliferation contributes to airway remodeling in severe asthma. Am J Respir Crit Care Med. (2007) 176(2):138–45. 10.1164/rccm.200607-1062OC 17463414 PMC1994213

[35] Boateng E Krauss-Etschmann S. miRNAs in lung development and diseases. Int J Mol Sci. (2020) 21(8):2765. 10.3390/ijms21082765 PMC7216056 32316149

[36] Perdomo C Campbell JD Gerrein J Tellez CS Garrison CB Walser TC Microrna 4423 is a primate-specific regulator of airway epithelial cell differentiation and lung carcinogenesis. Proc Natl Acad Sci USA. (2013) 110(47):18946–51. 10.1073/pnas.1220319110 24158479 PMC3839685

[37] Otto T Candido SV Pilarz MS Sicinska E Bronson RT Bowden M Cell cycle-targeting micrornas promote differentiation by enforcing cell-cycle exit. Proc Natl Acad Sci USA. (2017) 114(40):10660–5. 10.1073/pnas.1702914114 28923932 PMC5635871

[38] Martinez-Anton A Sokolowska M Kern S Davis AS Alsaaty S Taubenberger JK Changes in microrna and mrna expression with differentiation of human bronchial epithelial cells. Am J Respir Cell Mol Biol. (2013) 49(3):384–95. 10.1165/rcmb.2012-0368OC 23590309 PMC3824051

[39] Xiong T Du Y Fu Z Geng G. Microrna-145-5p promotes asthma pathogenesis by inhibiting kinesin family member 3a expression in mouse airway epithelial cells. J Int Med Res. (2019) 47(7):3307–19. 10.1177/0300060518789819 31264490 PMC6683905

[40] Morrisey EE Hogan BL. Preparing for the first breath: genetic and cellular mechanisms in lung development. Dev Cell. (2010) 18(1):8–23. 10.1016/j.devcel.2009.12.010 20152174 PMC3736813

[41] Haies DM Gil J Weibel ER. Morphometric study of rat lung cells. I. Numerical and dimensional characteristics of parenchymal cell population. Am Rev Respir Dis. (1981) 123(5):533–41. 10.1164/arrd.1981.123.5.533 7015935

[42] Wang Y Tang Z Huang H Li J Wang Z Yu Y Pulmonary alveolar type I cell population consists of two distinct subtypes that differ in cell fate. Proc Natl Acad Sci U S A. (2018) 115(10):2407–12. 10.1073/pnas.1719474115 29463737 PMC5877944

[43] Barkauskas CE Cronce MJ Rackley CR Bowie EJ Keene DR Stripp BR Type 2 alveolar cells are stem cells in adult lung. J Clin Invest. (2013) 123(7):3025–36. 10.1172/JCI68782 23921127 PMC3696553

[44] Desai TJ Brownfield DG Krasnow MA. Alveolar progenitor and stem cells in lung development, renewal and cancer. Nature. (2014) 507(7491):190–4. 10.1038/nature12930 24499815 PMC4013278

[45] Rock JR Randell SH Hogan BL. Airway basal stem cells: a perspective on their roles in epithelial homeostasis and remodeling. Dis Model Mech. (2010) 3(9–10):545–56. 10.1242/dmm.006031 20699479 PMC2931533

[46] Whitsett JA. Airway epithelial differentiation and mucociliary clearance. Ann Am Thorac Soc. (2018) 15(Suppl 3):S143–8. 10.1513/AnnalsATS.201802-128AW 30431340 PMC6322033

[47] Montoro DT Haber AL Biton M Vinarsky V Lin B Birket SE A revised airway epithelial hierarchy includes cftr-expressing ionocytes. Nature. (2018) 560(7718):319–24. 10.1038/s41586-018-0393-7 30069044 PMC6295155

[48] Rock JR Gao X Xue Y Randell SH Kong YY Hogan BL. Notch-dependent differentiation of adult airway basal stem cells. Cell Stem Cell. (2011) 8(6):639–48. 10.1016/j.stem.2011.04.003 21624809 PMC3778678

[49] Boers JE Ambergen AW Thunnissen FB. Number and proliferation of basal and parabasal cells in normal human airway epithelium. Am J Respir Crit Care Med. (1998) 157(6 Pt 1):2000–6. 10.1164/ajrccm.157.6.9707011 9620938

[50] Knight DA Holgate ST. The airway epithelium: structural and functional properties in health and disease. Respirology. (2003) 8(4):432–46. 10.1046/j.1440-1843.2003.00493.x 14708552

[51] Rawlins EL Okubo T Xue Y Brass DM Auten RL Hasegawa H The role of Scgb1a1+ clara cells in the long-term maintenance and repair of lung airway, but not alveolar, epithelium. Cell Stem Cell. (2009) 4(6):525–34. 10.1016/j.stem.2009.04.002 19497281 PMC2730729

[52] Song H Yao E Lin C Gacayan R Chen MH Chuang PT. Functional characterization of pulmonary neuroendocrine cells in lung development, injury, and tumorigenesis. Proc Natl Acad Sci USA. (2012) 109(43):17531–6. 10.1073/pnas.1207238109 23047698 PMC3491514

[53] Ouadah Y Rojas ER Riordan DP Capostagno S Kuo CS Krasnow MA. Rare pulmonary neuroendocrine cells are stem cells regulated by Rb, P53, and notch. Cell. (2019) 179(2):403–16 e23. 10.1016/j.cell.2019.09.010 31585080 PMC6782070

[54] Bloodgood RA. Sensory reception is an attribute of both primary cilia and motile cilia. J Cell Sci. (2010) 123(Pt 4):505–9. 10.1242/jcs.066308 20144998

[55] Kuek LE Lee RJ. First contact: the role of respiratory cilia in host-pathogen interactions in the airways. Am J Physiol Lung Cell Mol Physiol. (2020) 319(4):L603–19. 10.1152/ajplung.00283.2020 32783615 PMC7516383

[56] Park KS Wells JM Zorn AM Wert SE Laubach VE Fernandez LG Transdifferentiation of ciliated cells during repair of the respiratory epithelium. Am J Respir Cell Mol Biol. (2006) 34(2):151–7. 10.1165/rcmb.2005-0332OC 16239640 PMC2644179

[57] Singh G Katyal SL. Clara cell proteins. Ann N Y Acad Sci. (2000) 923:43–58. 10.1111/j.1749-6632.2000.tb05518.x 11193778

[58] Rostami MR LeBlanc MG Strulovici-Barel Y Zuo W Mezey JG O'Beirne SL Smoking shifts human small airway epithelium club cells toward a lesser differentiated population. NPJ Genom Med. (2021) 6(1):73. 10.1038/s41525-021-00237-1 34497273 PMC8426481

[59] Giangreco A Reynolds SD Stripp BR. Terminal bronchioles harbor a unique airway stem cell population that localizes to the bronchoalveolar duct junction. Am J Pathol. (2002) 161(1):173–82. 10.1016/S0002-9440(10)64169-7 12107102 PMC1850682

[60] Hong KU Reynolds SD Giangreco A Hurley CM Stripp BR. Clara cell secretory protein-expressing cells of the airway neuroepithelial body microenvironment include a label-retaining subset and are critical for epithelial renewal after progenitor cell depletion. Am J Respir Cell Mol Biol. (2001) 24(6):671–81. 10.1165/ajrcmb.24.6.4498 11415931

[61] Reynolds SD Giangreco A Power JH Stripp BR. Neuroepithelial bodies of pulmonary airways serve as a reservoir of progenitor cells capable of epithelial regeneration. Am J Pathol. (2000) 156(1):269–78. 10.1016/S0002-9440(10)64727-X 10623675 PMC1868636

[62] Gamez AS Gras D Petit A Knabe L Molinari N Vachier I Supplementing defect in club cell secretory protein attenuates airway inflammation in copd. Chest. (2015) 147(6):1467–76. 10.1378/chest.14-1174 25474370

[63] Mango GW Johnston CJ Reynolds SD Finkelstein JN Plopper CG Stripp BR. Clara cell secretory protein deficiency increases oxidant stress response in conducting airways. Am J Physiol. (1998) 275(2):L348–56. 10.1152/ajplung.1998.275.2.L348 9700096

[64] Tokita E Tanabe T Asano K Suzaki H Rubin BK. Club cell 10-Kda protein attenuates airway mucus hypersecretion and inflammation. Eur Respir J. (2014) 44(4):1002–10. 10.1183/09031936.00080913 24833761

[65] Laoukili J Perret E Willems T Minty A Parthoens E Houcine O Il-13 alters mucociliary differentiation and ciliary beating of human respiratory epithelial cells. J Clin Invest. (2001) 108(12):1817–24. 10.1172/JCI13557 11748265 PMC209466

[66] Rogers DF. Airway goblet cells: responsive and adaptable front-line defenders. Eur Respir J. (1994) 7(9):1690–706. 10.1183/09031936.94.07091678 7995400

[67] Plasschaert LW Zilionis R Choo-Wing R Savova V Knehr J Roma G A single-cell atlas of the airway epithelium reveals the Cftr-rich pulmonary ionocyte. Nature. (2018) 560(7718):377–81. 10.1038/s41586-018-0394-6 30069046 PMC6108322

[68] Okuda K Dang H Kobayashi Y Carraro G Nakano S Chen G Secretory cells dominate airway cftr expression and function in human airway superficial epithelia. Am J Respir Crit Care Med. (2021) 203(10):1275–89. 10.1164/rccm.202008-3198OC 33321047 PMC8456462

[69] Goldfarbmuren KC Jackson ND Sajuthi SP Dyjack N Li KS Rios CL Dissecting the cellular specificity of smoking effects and reconstructing lineages in the human airway epithelium. Nat Commun. (2020) 11(1):2485. 10.1038/s41467-020-16239-z 32427931 PMC7237663

[70] Prefontaine D Hamid Q. Airway epithelial cells in asthma. J Allergy Clin Immunol. (2007) 120(6):1475–8. 10.1016/j.jaci.2007.09.041 17980414

[71] Kouzaki H Tojima I Kita H Shimizu T. Transcription of interleukin-25 and extracellular release of the protein is regulated by allergen proteases in airway epithelial cells. Am J Respir Cell Mol Biol. (2013) 49(5):741–50. 10.1165/rcmb.2012-0304OC 23590308 PMC5455604

[72] Wang W Li Y Lv Z Chen Y Li Y Huang K Bronchial allergen challenge of patients with atopic asthma triggers an alarmin (Il-33, Tslp, and Il-25) response in the airways epithelium and submucosa. J Immunol. (2018) 201(8):2221–31. 10.4049/jimmunol.1800709 30185520

[73] Whetstone CE Ranjbar M Omer H Cusack RP Gauvreau GM. The role of airway epithelial cell alarmins in asthma. Cells. (2022) 11(7):1105. 10.3390/cells11071105 35406669 PMC8997824

[74] Barnig C Cernadas M Dutile S Liu X Perrella MA Kazani S Lipoxin A4 regulates natural killer cell and type 2 innate lymphoid cell activation in asthma. Sci Transl Med. (2013) 5(174):174ra26. 10.1126/scitranslmed.3004812 23447017 PMC3823369

[75] Hammad H Chieppa M Perros F Willart MA Germain RN Lambrecht BN. House dust mite allergen induces asthma via toll-like receptor 4 triggering of airway structural cells. Nat Med. (2009) 15(4):410–6. 10.1038/nm.1946 19330007 PMC2789255

[76] Bonnelykke K Sleiman P Nielsen K Kreiner-Moller E Mercader JM Belgrave D A genome-wide association study identifies Cdhr3 as a susceptibility locus for early childhood asthma with severe exacerbations. Nat Genet. (2014) 46(1):51–5. 10.1038/ng.2830 24241537

[77] Mortensen LJ Kreiner-Moller E Hakonarson H Bonnelykke K Bisgaard H. The Pcdh1 gene and asthma in early childhood. Eur Respir J. (2014) 43(3):792–800. 10.1183/09031936.00021613 23988763

[78] Toncheva AA Suttner K Michel S Klopp N Illig T Balschun T Genetic variants in protocadherin-1, bronchial hyper-responsiveness, and asthma subphenotypes in German children. Pediatr Allergy Immunol. (2012) 23(7):636–41. 10.1111/j.1399-3038.2012.01334.x 23050600

[79] Moheimani F Hsu AC Reid AT Williams T Kicic A Stick SM The genetic and epigenetic landscapes of the epithelium in asthma. Respir Res. (2016) 17(1):119. 10.1186/s12931-016-0434-4 27658857 PMC5034566

[80] Wiesner DL Merkhofer RM Ober C Kujoth GC Niu M Keller NP Club cell Trpv4 serves as a damage sensor driving lung allergic inflammation. Cell Host Microbe. (2020) 27(4):614–28 e6. 10.1016/j.chom.2020.02.006 32130954 PMC7305569

[81] Laing IA Hermans C Bernard A Burton PR Goldblatt J Le Souef PN. Association between plasma Cc16 levels, the A38g polymorphism, and asthma. Am J Respir Crit Care Med. (2000) 161(1):124–7. 10.1164/ajrccm.161.1.9904073 10619808

[82] Shijubo N Itoh Y Yamaguchi T Imada A Hirasawa M Yamada T Clara cell protein-positive epithelial cells are reduced in small airways of asthmatics. Am J Respir Crit Care Med. (1999) 160(3):930–3. 10.1164/ajrccm.160.3.9803113 10471621

[83] Oh JY Lee YS Min KH Hur GY Lee SY Kang KH Decreased serum club cell secretory protein in asthma and chronic obstructive pulmonary disease overlap: a pilot study. Int J Chron Obstruct Pulmon Dis. (2018) 13:3411–7. 10.2147/COPD.S174545 30425470 PMC6203108

[84] Zhu L An L Ran D Lizarraga R Bondy C Zhou X The club cell marker Scgb1a1 downstream of Foxa2 is reduced in asthma. Am J Respir Cell Mol Biol. (2019) 60(6):695–704. 10.1165/rcmb.2018-0199OC 30576223 PMC6543749

[85] Rajavelu P Chen G Xu Y Kitzmiller JA Korfhagen TR Whitsett JA. Airway epithelial Spdef integrates goblet cell differentiation and pulmonary Th2 inflammation. J Clin Invest. (2015) 125(5):2021–31. 10.1172/JCI79422 25866971 PMC4463206

[86] Dabbagh K Takeyama K Lee HM Ueki IF Lausier JA Nadel JA. Il-4 induces mucin gene expression and goblet cell metaplasia in vitro and in vivo. J Immunol. (1999) 162(10):6233–7. 10229869

[87] Danahay H Pessotti AD Coote J Montgomery BE Xia D Wilson A Notch2 is required for inflammatory cytokine-driven goblet cell metaplasia in the lung. Cell Rep. (2015) 10(2):239–52. 10.1016/j.celrep.2014.12.017 25558064

[88] Lora JM Zhang DM Liao SM Burwell T King AM Barker PA Tumor necrosis factor-alpha triggers mucus production in airway epithelium through an Ikappab kinase beta-dependent mechanism. J Biol Chem. (2005) 280(43):36510–7. 10.1074/jbc.M507977200 16123045

[89] Tyner JW Kim EY Ide K Pelletier MR Roswit WT Morton JD Blocking airway mucous cell metaplasia by inhibiting Egfr antiapoptosis and Il-13 transdifferentiation signals. J Clin Invest. (2006) 116(2):309–21. 10.1172/JCI25167 16453019 PMC1359039

[90] Sun L Ren X Wang IC Pradhan A Zhang Y Flood HM The Foxm1 inhibitor Rcm-1 suppresses goblet cell metaplasia and prevents Il-13 and Stat6 signaling in allergen-exposed mice. Sci Signal. (2017) 10(475):eaai8583. 10.1126/scisignal.aai8583 28420758

[91] Kuchibhotla VNS Starkey MR Reid AT Heijink IH Nawijn MC Hansbro PM Inhibition of beta-catenin/creb binding protein signaling attenuates house dust mite-induced goblet cell metaplasia in mice. Front Physiol. (2021) 12:690531. 10.3389/fphys.2021.690531 34385933 PMC8353457

[92] Ren X Shah TA Ustiyan V Zhang Y Shinn J Chen G Foxm1 promotes allergen-induced goblet cell metaplasia and pulmonary inflammation. Mol Cell Biol. (2013) 33(2):371–86. 10.1128/MCB.00934-12 23149934 PMC3554115

[93] Alagha K Bourdin A Vernisse C Garulli C Tummino C Charriot J Goblet cell hyperplasia as a feature of neutrophilic asthma. Clin Exp Allergy. (2019) 49(6):781–8. 10.1111/cea.13359 30710420

[94] Zeki AA Bratt JM Rabowsky M Last JA Kenyon NJ. Simvastatin inhibits goblet cell hyperplasia and lung arginase in a mouse model of allergic asthma: a novel treatment for airway remodeling? Transl Res. (2010) 156(6):335–49. 10.1016/j.trsl.2010.09.003 21078495 PMC2990975

[95] Chen M Lv Z Zhang W Huang L Lin X Shi J Triptolide suppresses airway goblet cell hyperplasia and Muc5ac expression via Nf-Kappab in a murine model of asthma. Mol Immunol. (2015) 64(1):99–105. 10.1016/j.molimm.2014.11.001 25466609

[96] Mishina K Shinkai M Shimokawaji T Nagashima A Hashimoto Y Inoue Y Ho-1 inhibits Il-13-induced goblet cell hyperplasia associated with Clca1 suppression in normal human bronchial epithelial cells. Int Immunopharmacol. (2015) 29(2):448–53. 10.1016/j.intimp.2015.10.016 26507166

[97] Horvath G Sorscher EJ. Luminal fluid tonicity regulates airway ciliary beating by altering membrane stretch and intracellular calcium. Cell Motil Cytoskeleton. (2008) 65(6):469–75. 10.1002/cm.20273 18435452

[98] Luk CK Dulfano MJ. Effect of Ph, viscosity and ionic-strength changes on ciliary beating frequency of human bronchial explants. Clin Sci (Lond). (1983) 64(4):449–51. 10.1042/cs0640449 6825413

[99] Shah AS Ben-Shahar Y Moninger TO Kline JN Welsh MJ. Motile cilia of human airway epithelia are chemosensory. Science. (2009) 325(5944):1131–4. 10.1126/science.1173869 19628819 PMC2894709

[100] Xiao C Puddicombe SM Field S Haywood J Broughton-Head V Puxeddu I Defective epithelial barrier function in asthma. J Allergy Clin Immunol. (2011) 128(3):549–56 e1-12. 10.1016/j.jaci.2011.05.038 21752437

[101] Hackett TL Singhera GK Shaheen F Hayden P Jackson GR Hegele RG Intrinsic phenotypic differences of asthmatic epithelium and its inflammatory responses to respiratory syncytial virus and air pollution. Am J Respir Cell Mol Biol. (2011) 45(5):1090–100. 10.1165/rcmb.2011-0031OC 21642587

[102] Post S Heijink IH Hesse L Koo HK Shaheen F Fouadi M Characterization of a lung epithelium specific E-cadherin knock-out model: implications for obstructive lung pathology. Sci Rep. (2018) 8(1):13275. 10.1038/s41598-018-31500-8 30185803 PMC6125431

[103] Turner J Roger J Fitau J Combe D Giddings J Heeke GV Goblet cells are derived from a Foxj1-expressing progenitor in a human airway epithelium. Am J Respir Cell Mol Biol. (2011) 44(3):276–84. 10.1165/rcmb.2009-0304OC 20539013

[104] Vieira Braga FA Kar G Berg M Carpaij OA Polanski K Simon LM A cellular census of human lungs identifies novel cell states in health and in asthma. Nat Med. (2019) 25(7):1153–63. 10.1038/s41591-019-0468-5 31209336

[105] Collison A Mattes J Plank M Foster PS. Inhibition of house dust mite-induced allergic airways disease by antagonism of microrna-145 is comparable to glucocorticoid treatment. J Allergy Clin Immunol. (2011) 128(1):160–7 e4. 10.1016/j.jaci.2011.04.005 21571357

[106] Mattes J Collison A Plank M Phipps S Foster PS. Antagonism of microrna-126 suppresses the effector function of Th2 cells and the development of allergic airways disease. Proc Natl Acad Sci USA. (2009) 106(44):18704–9. 10.1073/pnas.0905063106 19843690 PMC2773983

[107] Sharma A Kumar M Ahmad T Mabalirajan U Aich J Agrawal A Antagonism of mmu-Mir-106a attenuates asthma features in allergic murine model. J Appl Physiol (1985). (2012) 113(3):459–64. 10.1152/japplphysiol.00001.2012 22700801

[108] Liu Z Chen X Wu Q Song J Wang L Li G. Mir-125b inhibits goblet cell differentiation in allergic airway inflammation by targeting Spdef. Eur J Pharmacol. (2016) 782:14–20. 10.1016/j.ejphar.2016.04.044 27112664

[109] Su Y Wang J Zou J Han W Li S. Mir-330 regulates interleukin-13-induced Muc5ac secretion by targeting Munc18b in human bronchial epithelial cells. Int J Clin Exp Pathol. (2018) 11(7):3463–70. 31949724 PMC6962880

[110] Solberg OD Ostrin EJ Love MI Peng JC Bhakta NR Hou L Airway epithelial mirna expression is altered in asthma. Am J Respir Crit Care Med. (2012) 186(10):965–74. 10.1164/rccm.201201-0027OC 22955319 PMC3530212

[111] Marcet B Chevalier B Luxardi G Coraux C Zaragosi LE Cibois M Control of vertebrate multiciliogenesis by Mir-449 through direct repression of the Delta/notch pathway. Nat Cell Biol. (2011) 13(6):693–9. 10.1038/ncb2241 21602795

[112] Jardim MJ Dailey L Silbajoris R Diaz-Sanchez D. Distinct microrna expression in human airway cells of asthmatic donors identifies a novel asthma-associated gene. Am J Respir Cell Mol Biol. (2012) 47(4):536–42. 10.1165/rcmb.2011-0160OC 22679274

[113] Martinez-Nunez RT Rupani H Plate M Niranjan M Chambers RC Howarth PH Genome-wide posttranscriptional dysregulation by micrornas in human asthma as revealed by Frac-Seq. J Immunol. (2018) 201(1):251–63. 10.4049/jimmunol.1701798 29769273 PMC6013048

[114] Kivihall A Aab A Soja J Sladek K Sanak M Altraja A Reduced expression of Mir-146a in human bronchial epithelial cells alters neutrophil migration. Clin Transl Allergy. (2019) 9:62. 10.1186/s13601-019-0301-8 31798831 PMC6880603

[115] Mortaz E Alipoor SD Varahram M Jamaati H Garssen J Mumby SE Exosomes in severe asthma: update in their roles and potential in therapy. Biomed Res Int. (2018) 2018:2862187. 10.1155/2018/2862187 29854739 PMC5964496

[116] Srinivasan A Sundar IK. Recent updates on the role of extracellular vesicles in the pathogenesis of allergic asthma. Extracell Vesicles Circ Nucl Acids. (2021) 2:127–47. 10.20517/evcna.2021.03 34414402 PMC8372030

[117] Gupta R Radicioni G Abdelwahab S Dang H Carpenter J Chua M Intercellular communication between airway epithelial cells is mediated by exosome-like vesicles. Am J Respir Cell Mol Biol. (2019) 60(2):209–20. 10.1165/rcmb.2018-0156OC 30230353 PMC6376407

[118] Nagano T Katsurada M Dokuni R Hazama D Kiriu T Umezawa K Crucial role of extracellular vesicles in bronchial asthma. Int J Mol Sci. (2019) 20(10):2589. 10.3390/ijms20102589 PMC6566667 31137771

[119] Ax E Jevnikar Z Cvjetkovic A Malmhall C Olsson H Radinger M T2 and T17 cytokines alter the cargo and function of airway epithelium-derived extracellular vesicles. Respir Res. (2020) 21(1):155. 10.1186/s12931-020-01402-3 32560723 PMC7304225

[120] Bartel S La Grutta S Cilluffo G Perconti G Bongiovanni A Giallongo A Human airway epithelial extracellular vesicle mirna signature is altered upon asthma development. Allergy. (2020) 75(2):346–56. 10.1111/all.14008 31386204

[121] Gon Y Maruoka S Inoue T Kuroda K Yamagishi K Kozu Y Selective release of mirnas via extracellular vesicles is associated with house-dust mite allergen-induced airway inflammation. Clin Exp Allergy. (2017) 47(12):1586–98. 10.1111/cea.13016 28859242

